# S-layers at second glance? Altiarchaeal grappling hooks (hami) resemble archaeal S-layer proteins in structure and sequence

**DOI:** 10.3389/fmicb.2015.00543

**Published:** 2015-06-09

**Authors:** Alexandra K. Perras, Bertram Daum, Christine Ziegler, Lynelle K. Takahashi, Musahid Ahmed, Gerhard Wanner, Andreas Klingl, Gerd Leitinger, Dagmar Kolb-Lenz, Simonetta Gribaldo, Anna Auerbach, Maximilian Mora, Alexander J. Probst, Annett Bellack, Christine Moissl-Eichinger

**Affiliations:** ^1^Department of Internal Medicine, Medical University of GrazGraz, Austria; ^2^Department of Microbiology and Archaea Center, University of RegensburgRegensburg, Germany; ^3^Department of Structural Biology, Max Planck Institute of BiophysicsFrankfurt, Germany; ^4^Department of Biophysics, University of RegensburgRegensburg, Germany; ^5^Chemical Sciences Division, Lawrence Berkeley National LaboratoryBerkeley, CA, USA; ^6^Faculty of Biology, Ludwig-Maximilians-University of MunichMunich, Germany; ^7^Research Unit Electron Microscopic Techniques, Institute of Cell Biology, Histology and Embryology, Medical University of GrazGraz, Austria; ^8^Institute of Cell Biology, Histology and Embryology, Medical University of GrazGraz, Austria; ^9^Core Facility Ultrastructure, Analysis, Center for Medical Research Institute, Medical University of GrazGraz, Austria; ^10^Unité Biologie Moléculaire du Gene chez les Extrêmophiles, Departément de Microbiologie, Institut PasteurParis, France; ^11^Department of Earth and Planetary Science, University of California, BerkeleyBerkeley, CA, USA; ^12^BioTechMed-GrazGraz, Austria

**Keywords:** archaea, S-layers, archaeal cell surface appendages, hami, nano-grappling hooks, double-membrane, environmental transcriptomics, electron cryo-tomography

## Abstract

The uncultivated “*Candidatus* Altiarchaeum hamiconexum” (formerly known as SM1 Euryarchaeon) carries highly specialized nano-grappling hooks (“hami”) on its cell surface. Until now little is known about the major protein forming these structured fibrous cell surface appendages, the genes involved or membrane anchoring of these filaments. These aspects were analyzed in depth in this study using environmental transcriptomics combined with imaging methods. Since a laboratory culture of this archaeon is not yet available, natural biofilm samples with high *Ca*. A. hamiconexum abundance were used for the entire analyses. The filamentous surface appendages spanned both membranes of the cell, which are composed of glycosyl-archaeol. The hami consisted of multiple copies of the same protein, the corresponding gene of which was identified via metagenome-mapped transcriptome analysis. The hamus subunit proteins, which are likely to self-assemble due to their predicted beta sheet topology, revealed no similiarity to known microbial flagella-, archaella-, fimbriae- or pili-proteins, but a high similarity to known S-layer proteins of the archaeal domain at their N-terminal region (44–47% identity). Our results provide new insights into the structure of the unique hami and their major protein and indicate their divergent evolution with S-layer proteins.

## Introduction

In the course of evolution, nature has developed simple and fascinating solutions for various challenges. Particularly microbial life seems thus to harbor an enormous potential of exploitable biomaterial, such as enzymes and other biomolecules. These compounds are thought to prove very useful for diverse applications, in e.g., medicine, pharmacy, or industry (e.g., Beg et al., 2001; Hasan et al., 2006; Dutta and Kundu, 2014). However, the majority of naturally occurring exploitable biomaterial remains to be explored, because a substantial amount of microorganisms resist cultivation in the laboratory and thus escape detailed characterization of their metabolic potential and functional traits.

Cultivation-independent methods such as metagenomics enable scientists to directly access the genetic information of (entire) microbial communities. The sequence information retrieved can be used for assembly of near complete to complete genomes from key or underrepresented members of the communities (Tyson et al., 2004; Sharon and Banfield, 2013; Sharon et al., 2013). This information thus provides the basis for functional annotation of these novel microbial genomes. However, annotation of genes from lineages with only distant representatives is sometimes limited. Some cases have been reported in which approximately 50% of the predicted proteins could not be assigned a function (Baker et al., 2010; Kantor et al., 2013). Consequently, linking metagenomic data from uncultivated microorganisms with information retrieved by other molecular methods and/or imaging techniques in order to characterize such unknown predicted proteins is a promising approach. Imaging techniques, however, can currently not be conducted for highly complex microbial communities (e.g., those from soil) without substantial loss of information. Nevertheless, populations with low and simple diversity and uneven abundance of its members, such as the uncultivated acid mine drainage microbial community, can be studied in detail using a variety of these techniques, enabling researchers to link metagenomics to cellular characteristics (Comolli et al., 2009; Baker et al., 2010; Yelton et al., 2013; Comolli and Banfield, 2014).

For instance, genomes of ARMAN cells have been linked to their ultrastructure (Baker et al., 2006, 2010; Comolli et al., 2009); the latter also revealed that their cell wall is composed of a double membrane—a highly unusual feature in the domain of Archaea (Klingl, 2014). This feature was described to be shared only with a few other members in this domain, which are represented by the genus *Ignicoccus* (Rachel et al., 2002; Junglas et al., 2008), the *Methanomassiliicoccus* species (Dridi et al., 2012; Iino et al., 2013; Borrel et al., 2014) and the recently described “*Candidatus* Altiarchaeum hamiconexum” (formerly known as SM1 Euryarchaeon, Rudolph et al., 2001; Probst et al., 2014).

*Ca*. A. hamiconexum is a representative of the recently introduced euryarchaeal order “*Candidatus* Altiarchaeales,” a widespread group of uncultivated archaea thriving in subsurface aquifers (Probst et al., 2014). The tiny coccoid *Ca*. A. hamiconexum cells are washed up from the subsurface in extraordinarily pure biofilms (Henneberger et al., 2006; Probst et al., 2013). In a very recent publication, metagenomic sequencing data of *Ca*. A. hamiconexum biofilms were combined with isotopic-based lipidomics to reveal its autotrophic metabolism, which may be the basis for substantial carbon dioxide fixation in the subsurface. Lipidomics has further shown that the archaeon's double membrane is composed of core diether lipids with either two phytanyl chains or a combination of one phytanyl and one sesterpanyl chain (Probst et al., 2014). Anchored in this membrane, unique cell surface appendages called “hami” (singular: “hamus”; Moissl et al., 2005) were identified via ultrastructural analyses. Hundreds of these hami protrude from each cell and interlink with those of neighboring cells in order to form a biofilm (Probst et al., 2014). Each filament is assembled from three protein sub-filaments that are wound up to a barbed-wire-like structure and a distal grappling hook. This unique structure is supposed to be composed of one major protein species (Moissl et al., 2005; Probst et al., 2014)—similar to surface layer proteins (S-layer), which usually also consist of one or two protein species that assemble into a 2-dimensional array on cell surfaces (Sleytr et al., 1988; Eichler, 2003; Veith et al., 2009; Klingl et al., 2011; for a detailed review on archaeal S-layers see Klingl, 2014, same Research Topic). It was proposed that individual hami subunits are expressed in the cytoplasm, transferred through the inner membrane by the Sec-pathway and then assembled in the periplasmic space between inner and outer membrane (Probst et al., 2014). Although six genes were annotated as putative hamus subunit-encoding genes, the actual gene that is expressed for hamus formation *in vivo* has not been identified so far (Probst et al., 2014).

In this study a combination of -omic techniques with electron microscopy was applied in order to identify the *bona fide* gene sequence of the hamus subunit, shed light onto its phylogenetic evolution and further analyze its structure and the membrane in which it is anchored. Due to its barbed-wire-like structure and in particular its distal nano-hook, the hamus is considered an exploitable biomaterial and thus a tool for nanobiotechnology (Moissl-Eichinger et al., 2012), for which we provide the basis in this communication.

## Materials and methods

### Sampling and sampling processing

Archaeal biofilm samples were taken from the cold sulfidic spring, called Mühlbacher Schwefelquelle Isling (MSI), which is located in close vicinity to Regensburg, Germany (Rudolph et al., 2004). The biofilms were harvested from double-opened flasks, which were incubated for 2–3 days in the water-flow of the spring, approx. 30 cm below the surface. The flasks were equipped with a polyethylene net, which proved useful to catch upwelling biofilm-pieces from the spring-water. After the incubation period, the entire flask was closed under water using rubber stoppers and transported on ice to the laboratory, where the samples were immediately processed (Probst et al., 2013).

### TOF-SIMS (time of flight secondary ion mass spectrometry) chemical imaging

Archaeal biofilms were washed from the nets, and free-floating biofilm pieces were collected onto on gold-plated screens (hole 100 μm, G225G1, Plano GmbH, Wetzlar, Germany). Samples were immediately dried and the gold-coated aperture disks were placed onto silicon wafers and affixed along the edges with adhesive tape, with care to avoid contact with the biofilm.

Chemical imaging was performed with a modified commercial reflectron-type time-of-flight secondary ion mass spectrometer (TOF.SIMS V; IonTOF, Germany). Mass-selected Bi^+^_3_ ions with 25 keV kinetic energy impacted the sample surface at 45° with respect to the surface normal. Ejected cationic and anionic chemical species were collected in separate analyses. Time-of-flight secondary ion mass spectrometry (TOF-SIMS) spectra were acquired with Bi^+^_3_ pulses in high current bunched mode, over an area of 500 μm × 500 μm, with a 256 pixel × 256 pixel raster scan at a repetition rate of 2.5 kHz, and secondary ions were extracted with a 10 μs long extraction −2000 V (positive ion mode) or +2000 V (negative ion mode) pulse. Electron charge compensation was not used.

### Electron cryo-tomography

Biofilm samples were centrifuged at 16,000 × g using an Eppendorf 5415 C table top centrifuge, and cell pellets were resuspended in an equal volume of KPH buffer (0.7 mM NaCl, 0.1 mM MgCl_2_, 1.6 mM CaSO_4_, 1 mM HEPES; the pH was adjusted with NaOH to 6.5; Moissl et al., 2005). Cellular suspensions were mixed with an equal amount of 10 nm colloidal protein-A gold (Aurion, Wageningen, The Netherlands), and 3 μl of this mixture were added to a glow-discharged Quantifoil (Quantifoil, Großlöbichau, Germany) grid, blotted for 3–5 s and plunged into liquid ethane.

Samples were transferred frozen into a Polara G2 Tecnai transmission electron microscope (FEI, Hillsboro, USA) operated at 300 kV. The TEM was equipped with a Gatan 4 × 4 k charge-coupled device (CCD) camera or a K2-Summit dierect electron detector as well as a Tridiem energy filter (Gatan Inc., Pleasanton, USA). Images were recorded using a magnification of 34,000 × on the CCD and 41,000 x on the K2 summit, corresponding to a pixel size of 0.6 or 0.54 in the final image, respectively. Zero loss filtered tomographic tilt series were collected in a range of −60° to +60°, at increments of 2°–2.5° and a defocus of 6–10 μm using the Gatan Digital Micrograph Latitude software (Gatan Inc., Pleasanton, USA) The maximum cumulative dose was 150 e^−^/A^2^.

For electron tomography at room temperature, samples were high-pressure frozen and embedded in epon resin as described in Perras et al. (2014). Tilt series were recorded at 120 kV on a JEOL JEM-2100, equipped with a LaB_6_ cathode and a 2 × 2 k F214 fast scan CCD camera (TVIPS, Gauting, Germany) in a range of −60° to +60° in steps of 2°, a defocus of 3–6 μm and a magnification of 14,000 ×, which equals a pixel size of 1 nm in the final micrograph. Tilt series were reconstructed into tomograms with the IMOD software (Kremer et al., 1996), using weighted backprojection or SIRT and displayed in 3 dmod (IMOD).

### Ultrastructural analysis using transmission electron microscopy (TEM) and scanning electron microscopy (SEM)

For analysing the cell surface appendages, the unfixed, purified hami were deposited on a carbon-coated copper grid and negatively stained with uranylacetate [2% (w/v), pH 4.5]. The samples were examined with a CM12 transmission electron microscope (FEI Co., Eindhoven, The Netherlands) operated at 120 kV. All images were digitally recorded using a slow-scan CCD camera that was connected to a computer with TVIPS software (TVIPS GmbH, Gauting, Germany). Scanning electron microscopy was performed as described in Probst et al. (2014).

### Particle characterization within the cells

For element analysis of the particles within coccoid cells, *Ca*. A. hamiconexum biofilm flocks were embedded in TAAB embedding resin (TAAB, Aldermaston, UK) and thin sectioned as described in Milić et al. (2015) followed by staining with platinum blue and lead citrate. Energy Filtered TEM (EFTEM) was performed with a Gatan GIF Quantum 963 energy filter using an FEI Tecnai 20 microscope at 120 kV acceleration voltage. To visualize the elemental distributions, elemental maps were made using the three window method at the standard losses provided by Gatan Digital Micrograph software (see also Teubl et al., 2014).

Energy Dispersive X-Ray spectroscopy was performed using an Edax silicum type ultrathin unit (SUTW) detector, as described in Milić et al. (2015); the corresponding images were made with scanning transmission EM using a High Angle Annular Dark Field detector (HAADF).

### Purification of hamus filaments and antibody generation

For the production of hamus-specific antibodies for protein analyses and structural investigations, hamus filaments were released from the archaeal cell surface. The purification procedure, as well as the production of hamus-specific antibodies, has been described elsewhere (Probst et al., 2014). In brief, the archaeal biofilm cells were lysed using 0.1% (w/v) sodium dodecyl sulfate (SDS) and cell debris was removed via subsequent centrifugation and sucrose-gradient centrifugation steps.

### Denaturating SDS-PAGE analysis and western blot

Hamus filaments were purified as described above and mixed with protein loading dye [Tris/HCl pH 7.5, 60 Mm; Glycerol 10% (v/v); SDS 2% (w/v); bromphenol blue 0.01% (w/v); 2-mercaptoethanol 5% (w/v)] and heated for 30 min in boiling water. Afterwards, proteins were separated via SDS-PAGE (Laemmli, 1970) using a Mini Protean 3 Cell [Bio-Rad Laboratories Inc., Munich; 12.5% (w/v) polyacrylamide linear gradient gel], at 15 mA followed by a higher current of 30 mA until the dye front reached the bottom of the gel.

The separated proteins were afterwards semi-dry-blotted onto a Roti®-PVDF (polyvinylidene fluoride) membrane (Carl Roth GmbH + Co. KG, Karlsruhe, Germany) using a semidry transfer cell instrument (Bio-Rad, Munich, Germany) operated at 16 V for 1 h. Blocking was performed by incubation of the membrane in Tris buffered saline [including Tween 200.01% (v/v), 3% milk powder (w/v); TBST-B] overnight. After a washing step with Tris buffered saline [including Tween 200.01% (v/v); TBST], the primary antibody (anti-hamus) was applied (1:5,000 dilution in TBST-B) and incubated for 3 h under agitation. The membrane was washed using TBST and the secondary antibody [anti-chicken coupled with horseradish peroxidase (1:1,000 in TBST-B; Sigma-Aldrich Chemie GmbH, Munich, Germany)], was applied for 2 h]. The reaction was visualized by applying a 3-amino-9-ethylcarbazole solution [20 mg of 3-amino-9-ethylcarbazole dissolved in 1 ml ethanol p.a., followed by mixing with 50 ml of potassium acetate, pH 5, 20 mM, 100 μl of triton X-100, 10% (v/v) and 10 μl of H_2_O_2_] after another washing step.

### Liquid chromatography mass spectrometry of peptides

For identification of peptides in the band showing positive reaction in the western blot analysis, the corresponding band in the SDS-PAGE was cut out and trypsin digested. Obtained peptides were then subjected to liquid chromatography coupled to tandem mass spectrometry (LC-MS/MS). HPLC was carried out using a Ultimate3000 RSLC nano-HPLC System (Thermo Fisher Scientific; at the facilities of Prof. Dr. R. Deutzmann, University of Regensburg) with a reversed phase chromatography analytical column (ReproSil Pur 120 C18-AQ, 75 μm × 25 cm). The mobile phase consisted of a linear gradient containing 0.1% (v/v) formic acid (eluent A) and 80% (v/v) acetonitrile, 0.1% (v/v) formic acid (eluent B). HPLC was coupled on-line to a maXis plus UHR-QTOF system (Bruker Daltonics) via nano-electrospray source and up to five most abundant precursors selected for fragmentation by collisional induced dissociation (CID). Identification of the obtained peptide mass fingerprints was performed by genome database searching using the PeptideMass software (Wilkins et al., 1997).

### Fluorescence immuno-labeling

For immuno-staining, the archaeal biofilms were fixed with paraformaldehyde (5%; v/v) at room temperature (1 h) and washed three times with PBS (phosphate buffered saline). The cell suspension was applied into a well of a gelatine-coated slide [P. Marienfeld KG, Lauda-Koenigshofen, Germany; slides were dipped into a 0.01% (w/v) gelatine solution and dust-free air-dried]. The dried cells were afterwards incubated in 16 μl of PBST [+0.1% (w/v) SDS] at 37°C and the PBST buffer was replaced with 16 μl PBST buffer containing the anti-hamus IgG (Davids biotechnology, Regensburg, Germany; dilution 1:2,000; 1 h, 37°C). Subsequently, the slide was washed 15 min in PBST [+0.1% (v/v) SDS], rinsed with H_2_O and air dried. The secondary antibody (goat anti-chicken, Cy3-labeled; 0.64 mg/ml, Sigma Aldrich, Germany; dilution 1:500) was added and incubated for 1 h at 37°C. After washing two times with PBST [+0.1% (v/v) SDS], the slide was rinsed with H_2_O again, DAPI counterstained and analyzed by fluorescence microscopy (Olympus BX53F, Hamburg, Germany) with epifluorescence equipment and the respective imaging software (cellSens, Olympus).

### Transcriptomic analysis of hamus gene(S)

The presence or absence of specific hamus subunit transcripts was tested via specific mRNA detection in archaeal biofilm samples (see Supplementary Table S1, containing list of genes and primers). Three hamus subunits (i.e., MSIBFv1_A2980002, MSIBFv1_A2020020, MSIBFv1_A3210004; deposited in the European Nucleotide Archive; accession code: PRJEB6121; Probst et al., 2014) were examined in detail.

RNA was isolated using the PowerBiofilm™ RNA Isolation Kit (Mobio Laboratories Inc., Carlsbad, USA), following the manufacturers' instructions (DNA digestion was performed for 30 min). DNAse treatment was repeated after precipitation of nucleic acids. Contamination of the RNA extraction by residual DNA was excluded by PCR amplification, using a universal archaeal forward primer combined with a reverse primer binding in a non-transcribed region (e.g., primer: 344aF and 64R-23S, sequences in Supplementary Table S1). Samples showing no positive PCR signals were assumed to be free of contaminating DNA and were further processed. RNA was reverse-transcribed into cDNA (QuantiTect Rev. Transcription Kit, Qiagen, Hilden, Germany). Transcripts were amplified using specific primers, which were designed using the web tool Primer3v.0.4.0 software (http://biotools.umassmed.edu/bioapps/primer3_www.cgi; parameters: product size: optimum full length of gene, GC% 40–60%, annealing temperature: 60°C optimum). To confirm the specificity, primers were searched (Altschul et al., 1990) against NCBI NR and against the *Ca*. A. hamiconexum metagenome (Probst et al., 2014). The designed primer pairs (see Supplementary Table S1) were used individually using cDNA as a template and tested for amplification success (denaturation time: 5 min 95°C; 30 cycles: 45 s 94°C, 45 s 60°C, 130 s 72°C; final elongation: 10 min 72°C). Positive PCR products were purified (HiYield® Gel/PCR DNA Fragments Extraction Kit; Süd-Laborbedarf GmbH, Gauting, Germany) and Sanger-sequenced (LGC Genomics GmbH, Berlin, Germany). Experiments were carried out in duplicates.

### Hamus protein analysis, structure determination, and phylogenetic tree reconstruction

The trans-membrane region of the hamus protein was predicted by TMHMM v2.0 (http://www.cbs.dtu.dk/services/TMHMM/). The protein characteristics were analyzed using GenScript's Peptide Property Calculator (https://www.genscript.com/ssl-bin/site2/peptide_calculation.cgi) and by NetNGlyc 1.0 (http://www.cbs.dtu.dk/services/NetNGlyc/).

Secondary structure was predicted using PSIPRED (http://bioinf.cs.ucl.ac.uk/psipred/), alignment of the sequences was performed using the multi-sequence alignment program MAFFT (http://mafft.cbrc.jp/alignment/server/). PSIPRED prediction was combined with a fold recognition search using pGenThreader (Lobley et al., 2009).

Hamus protein region 5–81 was searched against the NCBI database (blastp, Altschul et al., 1990). The 50 mostly related protein sequences were retrieved and used for tree (Neighbor Joining and Maximum Likelihood) reconstruction via MEGA 6 (Tamura et al., 2013).

## Results

### The *Ca*. A. hamiconexum double membrane is composed of glycosyl-archaeol species

In positive ion TOF-SIMS spectra, several notable mass spectral peaks were observed in the high mass range [700–1,200 atomic mass units (amu) and around 2,000 amu]. Most prominent of these mass peaks is a cluster of peaks centered about ~1,000 amu, which was assigned to sodiated diglycosyl archaeol (Figure S1). 16 mass units lower and higher of the sodiated lipid peaks were additional clusters of peaks which could either reflect lithiated and potassiated variants, or, for the m/z ~1,016 peaks contribution from a core hydroxyarchaeol with sodiated diglycosyl polar head group. Minor contributions of sodiated triglycosyl archaeol and monoglycosyl archaeol were also detected at m/z 1,162 and 838, respectively.

In addition to the salt adducts of the mono-, di- and tri-glycosyl lipids, a significant contribution from m/z ~1,070 was observed. From LC/MS/MS data of additional samples of the SM1 biofilm, this lipid was assigned to a diglycosyl diether lipid with one C_20_ hydrocarbon chain and one C_25_ hydrocarbon chain.

In the mass range where dimers or tetraether species would occur, several groups of peaks could also be observed, albeit at relatively low intensity (Figure S2). Prominent among these species was a cluster of peaks with a maximum peak intensity observed at m/z 1,974.5 and 2,006.5 amu. M/z 1,974.5 was found too low in mass to correspond to a simple sodiated dimer of the diglycosyl lipid (strongest isotope peak would appear at m/z 1,977.6), and too high in mass to correspond to a sodiated diglycosyl dialkyl tetraether (which would have its strongest isotope peak at m/z 1,973.5). Based on the strong sodiated lipid contribution in the diglycosyl diether lipid-related peaks, it was assumed that the peaks represented a sodiated species. With this assumption, one possible structure could be a sodiated *trialkyl* tetraether lipid with four total glycosyl groups on the ends (Figure S2). It is not clear whether this species is native to the biofilm or a result of a dimerization process that occurs during the ion sputtering event.

### Particles localized inside and outside of the *Ca*. A. hamiconexum cells reveal different elemental composition, indicating the presence of sulfur and phosphor deposits within the cells

**Figure 1:**
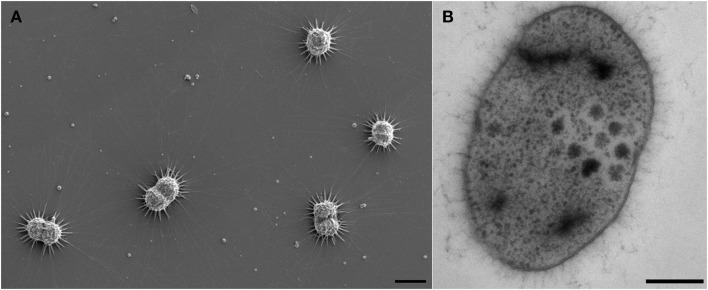
**Electron micrographs of** ***Ca.*** **A. hamiconexum cells within the biofilm**. **(A)** Scanning electron micrograph of dividing cells in the biofilm, showing particles close to the hami and the cells. Bar: 1 μm. **(B)** Thin-section of one cell, showing dark-stained areas. Bar. 200 nm.

TOF-SIMS analysis revealed inorganic species embedded in the biofilm, containing Na, K, Ca, Mg, and Fe (positive ion mode TOF-SIMS; Figure S3). Ca^2+^ and Mg^2+^ appeared to be concentrated within particles in the biofilm, which may indicate the formation of insoluble mineral carbonates. As revealed by scanning electron microscopy, such particles were easily visible in the preparations (Figure 1A), and many particles were located in close vicinity or even touched by the hami of highly actively dividing cells (Figure 1A).

Electron microscopic images of ultrathin-sectioned cells showed that most of the cells exhibited dark inclusions within their cells (Figure 1B). Such dense areas were examined using energy dispersive X-ray spectroscopy and were identified containing most likely phosphor and sulfur (in some cells) (data not shown). This result was further confirmed using EFTEM (Figure S4).

### Hamus-filaments are three-dimensional structures and span both membranes

**Figure 2:**
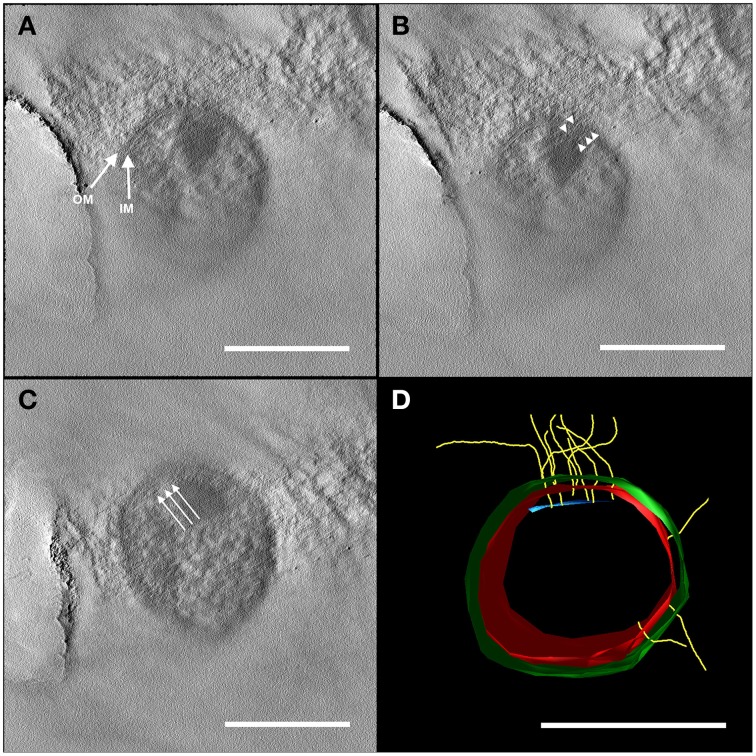
**Electron tomography of a 50 nm section of high-pressure frozen and freeze substituted cells**. The tilt series was carried out at 120 kV from −60 to +60° with an increment of 2° and a final magnification of 14,000 x. The reconstruction of the tomogram was performed with IMOD. In selected virtual slices, several structures were indicated. **(A)** The final tomogram revealed the inner (IM) and outer membrane (OM) with a thickness of about 6 nm in each case. The overall thickness of inner and OM together with the periplasm was 44 nm. Therefore, the periplasm has a width of about 32 nm. **(B)** Further, two hami filaments with a diameter of approximately 8 nm were indicated spanning both the IM and OM (arrow heads). **(C)** Underneath the IM, another layer could be recognized (arrows). As the resolution of the tomogram is quite low, this could either be kind of an anchoring structure/mechano sensor or a preparation/reconstruction artifact as it was just visible in a very low number of the virtual slices of the tomogram. With this tomogram, a model (segmentation) was constructed **(D)** illustrating the IM (red) and OM (green), the hami filaments (yellow) and the supposed anchoring-associated structure (blue). For simplicity, the membranes were illustrated as a monolayer. Scale bars: 500 nm.

Preliminary results of electron tomography from entire *Ca*. A. hamiconexum cells after high-pressure-freezing, freeze substitution and epon embedding resulted in a cell wall model (Figure 2). Both the inner and the outer membrane were visualized in a 50 nm section through the cell (Figure 2; Supplementary Movies 1, 2). Filamentous structures (8 nm in diameter), most likely representing the hami, passed through both membranes. At the cytoplasmic end of some of the filaments, an electron dense structure could be detected, representing a potential anchorage-associated structure of the filaments.

**Figure 3:**
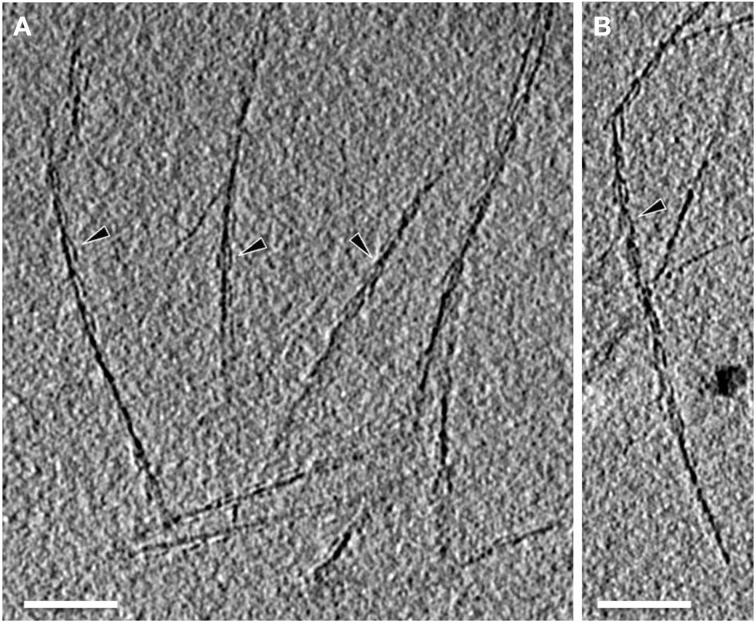
**Electron cryo-tomography of the hami**. Tomographic slices through hami emanating from the cell surface. Hami occur as single barbed wire-like filaments (**A,B**; black arrowheads). Scale bar: 100 nm.

**Figure 4:**
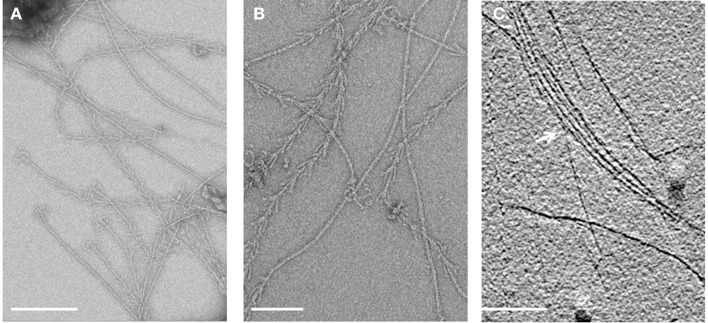
**Electron micrographs of isolated hami**. **(A,B)** Overview of negatively stained hami with visible triple grappling hooks. The typical barbed-wire structure of the filament is shown, next to plain (probably) stretched filaments. Electron cryo-tomography shows the bundling of the hami (**C**, white arrow). Scale bar: 500 nm.

Electron cryo-tomography of hami several μm away from the cell surface (Figure 3) revealed the typical barbed-wire structure as seen in micrographs of negatively stained samples (Figure 4A, see also: Moissl et al., 2005; Perras et al., 2014; Probst et al., 2014). In the tomograms, the hami showed a repeating pattern of prickle triplets at an average center-to-center distance of 47 nm. Each prickle revealed an average length of 36 nm and emanated from the backbone of the filament. However, hami missing the typical barbed-wire pattern were also observed (Figures 4A,B). They were partially plain filaments and could be identified as hami by their dimensions and typical hook (Figure 4A).

Apart from individual hamus filaments, bundles of such filaments were found (Figure 4C), suggesting that they are capable of forming super-filaments that are interlinked by the prickles. Tomograms of the cell body showed a plethora of filaments emanating from the cells (Supplementary Movies 3, 4). Most of these filaments showed the barbed wire-like structure typical for hami. Due to the thickness of the cell body, filaments passing the membranes could not be clearly resolved.

### The major hamus subunit protein is encoded by one of six homolog genes

**Figure 5:**
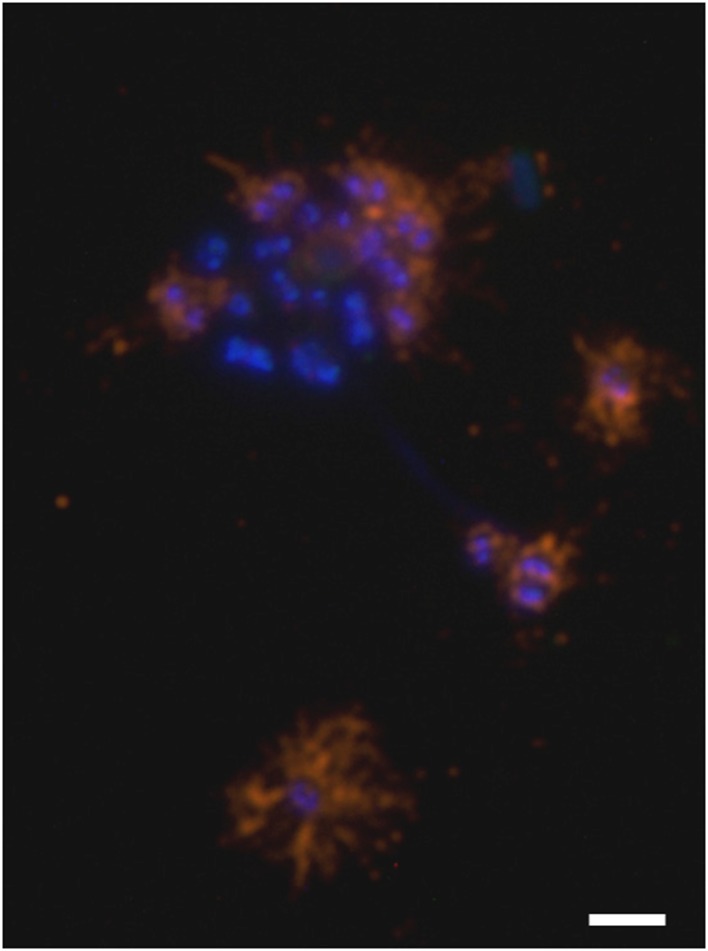
**Immuno-staining of cells embedded in the archaeal biofilm**. DNA is stained blue (DAPI) and hami were visualized using a CY3-labeled anti-hamus antibody (orange). The hami formed a halo around the cell. Scale bar: 2 μm.

Purified hami were found to be composed of one major protein (“120 kDa protein”; Moissl et al., 2005). The antibodies generated against purified hamus filaments showed a strong and specific reaction against the surfaces of coccoid *Ca*. A. hamiconexum cells within the biofilms (Figure 5). No signal was obtained from (filamentous) bacteria, occasionally enclosed in the biofilm. The same antibodies were used for a Western blot immuno-assay, showing a clear, strong signal appearing on the gel—the previously identified “120 kDa protein” (Figure S5; Moissl et al., 2005). The LC-MS/MS fingerprint from this protein was compared with the metagenomic information of *Ca*. A. hamiconexum retrieved during a recent study (Probst et al., 2014). Six homologous open reading frames (ORFs) potentially encoding for the major hamus protein were identified within the metagenomic data set. Supported by transcriptomic data (the retrieved RNA sequence revealed 100% identity in sequence and length), ORF MSIBFv1_A321004 was identified to code for the transcribed hamus subunit protein. This ORF was located on a relatively small contig (~5,600 nucleotides), containing only five ORFs in total. Transcription of the other homologous hamus genes could not be proven.

### The hamus protein carries glycosylation sites and an S-layer-like N-terminal region

The identified hamus ORF MSIBFv1_A321004 coded for an acidic, soluble 97 kDa protein (hydrophobicity: −0.28, PI 5.18) with major components Gly (9.71%) and Thr (9.49%). A sec-signal peptide was predicted [amino acid (aa) 1-27; Probst et al., 2014], and in the same region of the gene, in congruence, TMHMM predicted a transmembrane helix (aa: 7-29). This sequence was predicted to be hydrophobic and charged positively (pI: 10.51). At least seven potential glycosylation sites were proposed for the hamus subunit protein. Thus, the discrepancy between the protein mass estimated from SDS-PAGE (120 kDa) and the gene-predicted mass (97 kDa) resulted most likely from post-translational glycosylation of the protein (Moissl et al., 2005).

The hamus subunit gene (ORF MSIBFv1_A321004) shared the contig with four additional ORFs (Figure S6), encoding for two proteins of unknown function, a glutamate-tRNA-ligase (closest related protein from *Methanobacterium formicicum*, DSM3637) and an acylphosphatase, with highest similarity to *Korarchaeum cryptofilum* (strain OPF8) acylphosphatase. One of the unknown proteins belongs to the TraB family, the other shows partial similarities (32%) to a *Sulfolobus solfataricus* (strain ATCC 33909) putative UDP-N-acetylglucosamine-dolichyl-phosphate N-acetylglucosamine-phosphotransferase.

Regarding the complete hamus subunit protein sequence, NCBI NR blastp search (Altschul et al., 1990) revealed no homologous sequences. However, three different architectures to be remotely related to parts of the protein sequence were revealed by the conserved Domain Architecture Retrieval Tool (http://www.ncbi.nlm.nih.gov/Structure/lexington/docs/cdart_about.html): Archaeal S-layer proteins, hypothetical proteins and acetyl-transferases. Patterns attributed to archaeal S-layer proteins were observed mainly at the N-terminal region of the protein [“S_layer_N”: S-layer like family, N-terminal region (pfam 05123, aa 5-81) and “S_layer_MJ”: S-layer protein, MJ0822 family (TIGR 01564, aa 5-98)]. Closest relatives were found to be N-terminal regions of S-layer proteins from *Methanotorris igneus* (WP_013799875.1; 47% identity, *e*-value: 6e-05; 56.6 bits), *Methanothermococcus thermolithotrophicus* (CAC83952.2; 44% identity, *e*-value 5e-04, 53.9 bits) and *Thermococcus eurythermalis* (AIU70131.1; 45%; *e*-value: 7e-04; 53.5 bits). In contrast to these and other known S-layer proteins, the hamus subunit-protein did not exhibit a typical S_layer_C- terminus pattern.

### The hamus protein exhibits a prominent β-sandwich fold and thus structurally resembles typical archaeal S-layers

The secondary structure prediction program PSIPRED identified about 56 beta strands connected by loops and flanked by helical segments in some cases (Figure S7). The first 110 amino acids showed sequence homologies to S-layer proteins from methanogenic Archaea (*Methanococcus voltae*, *Methanococcus maripaludis*, *Methanothermococcus okinawensis*, and *Methanocaldococcus vulcanius*). None of these homologous S-layer proteins have yet been investigated structurally and they do not show any sequence identity to solved structures of S-layer proteins, from bacterial *Clostridium difficile* [Protein Data Bank (pdb) entry 3cvz, (Fagan et al., 2009)], *Clostridium thermocellum* (pdb entry 4qvs) or archaeal S-layer proteins from *Methanosarcina acetivorans* (pdb entry 3u2h, 1l0q; Arbing et al., 2012). Therefore, it was not possible to find a suitable template for reasonable homology modeling just by using a BLAST algorithm.

Putative structural conservation patterns compared to the archaeal S-layer proteins were investigated by applying the multi-sequence alignment program MAFFT in combination with the topology prediction provided by PSIPRED (pdb entry 3u2h). A parallel overlap between sequence and topology conservation to the DUF1608 domain in the S-layer protein from *M. acetivorans* was identified, when only parts of the hamus protein sequence (Ala334-Asp665) were searched. The *M. acetivorans* S-layer protein MA0829 comprises 671 aa and, similar to the hamus protein, has an N-terminal signal peptide. In addition, it contains the tandem-duplicated DUF1608 domains exclusively found in methanogenic Euryarchaeota.

In a second step, a PSIPRED prediction combined with a fold recognition search (pGenThreader) was performed, which can be applied to individual protein sequences. Three hits were indicated with a p-score of 10^−8^ and an overall coverage of more than 50%. Interestingly, none of the proposed proteins were associated with S-layer proteins: (1) a cytoplasmic response regulator of two-component system, which controls heparin and heparan sulfate acquisition and degradation in the human gut symbiont *Bacteroides thetaiotaomicron* (pdb entry 4a2l; Lowe et al., 2012), (2) a xyloglucanase from *C. thermocellum* (pdb entry 2cn3, Martinez-Fleites et al., 2006), and (3) a human DNA-damage binding protein (pdb entry 3ei3; Scrima et al., 2008).

**Figure 6:**
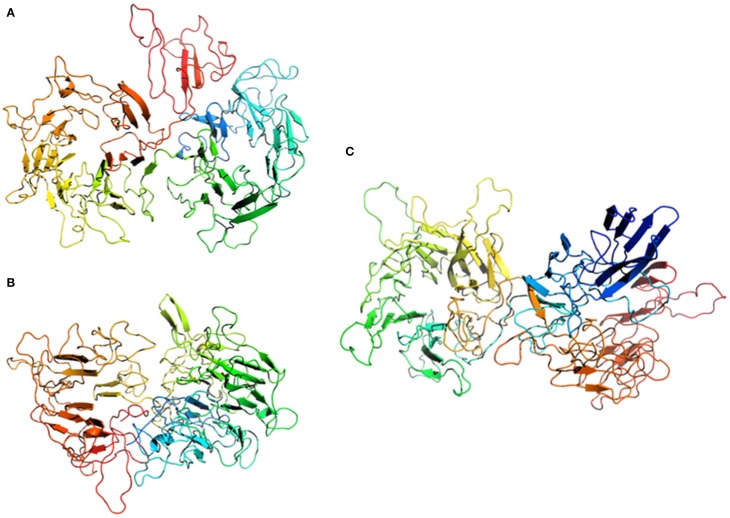
**Homology modeling of the Hami sequence on (A) a cytoplasmic response regulator of two-component system (pdb entry 4a2l), (B) a xyloglucanase from** ***Clostridium thermocellum*** **(pdb entry 2cn3), and (C) a human DNA-damage binding protein (pdb entry 3ei3)**. The models are displayed as cartoons colored in spectrum mode with the N-terminal region in blue and the C-terminal region in red.

We have performed homology modeling based on the first three hits of pGenThreader (Figure 6). In all three models the first 110 aa were removed. Two of the models (Figures 6A,C) show a propeller-like assembly reminiscent of a WD40 repeat. Although not being suitable for homology modeling, also the lower ranking hits proposed by pGenTHREADER revealed a consistent picture with an always returning motif of the WD40 propeller domain, which is often seen in S-layer proteins (Veith et al., 2009; Klingl et al., 2011) and represents one of the most conserved domains for protein-protein interaction. In the model of the hamus protein based on the template of the DNA-damage binding protein (pdb entry 3ei3) this motif would cover the region from aa 230-600, while in the model based on the template of the xyloglucanase from *C. thermocellum* (pdb entry 2cn3) the WD40 like array would correspond to aa 440-800.

Phylogenetic analyses of the hamus protein N-terminus region (aa 1-98) revealed a distinct position within the Euryarchaeota as displayed in Figure S8. Maximum Likelihood and Neighbor Joining analyses revealed similar results, supporting the hamus subunit protein N-terminus localization as a separate branch (Figure S8).

## Discussion

The filamentous cell surface appendages of the cold-loving, uncultivated SM1 Euryarchaeon, *Ca*. A. hamiconexum (Probst et al., 2014) are composed of one major protein species. The sequence of this hamus subunit protein did not show any homologies to currently known proteins involved in microbial fiber-, pili-, flagella-, or archaella formation, but showed similarities to known archaeal S-layer proteins: Besides a typical S-layer N-terminus pattern, the hamus subunit protein was found to be slightly acidic and most likely highly glycosylated, similar to S-layer proteins from e.g., *Acidianus ambivalens* and *Metallosphaera sedula* (Veith et al., 2009). In addition, the hamus subunit protein revealed a prominent beta sheet topology and thus might be primed for self-assembly (Makabe et al., 2006). Previous modeling of beta-rich structures has shown that conformational diversity over a large number of repeats can lead to significantly different self-assemblies therein (such as the formation of fibrils, films, and ribbons) and that their final structure is determined by the way inherent flexibility is maintained via beta-sheet twists and bends (Makabe et al., 2006).

Although there is a remarkable lack of sequence similarity between archaeal S-layer proteins, which also limited the modeling possibilities, β-sandwich structures are obviously typical features of euryarchaeal S-layers (Arbing et al., 2012) and of S-layers from more distantly related archaea, such as Sulfulobales (Veith et al., 2009). Moreover, it is likely that the β-sandwich domains are structurally related to other proteins associated with enveloping functions not only in archaea but also in bacteria, fungi, and viruses, emphasizing the general principle and self-assembly nature of beta-sheet rich proteins (Arbing et al., 2012). In general, S-layer proteins (with a size range between 40 and 210 kDa; Sleytr and Sára, 1997; Sleytr et al., 1997; König et al., 2007) are able to self-assemble into different lattices of oblique, tetragonal or hexagonal architecture—and can even exhibit complex, unusual structures, such as the tetrabrachion of *Staphylothermus marinus* (Engelhardt and Peters, 1998). This S-layer associated protein complex possesses umbrella-like thread morphology and distal branched quadrupled arms (Peters et al., 1995). The S-layer itself depicts p4-symmetry and the long stalks, which anchor the protein in the cytoplasmic membrane, form a 70 nm wide pseudoperiplasm. Furthermore, this membrane anchor is associated with a protease (STABLE; Peters et al., 1995), which might have a metabolic function for this species. Although the resemblance of tetrabrachion and hami appears striking, no similarity between both proteins could be shown on structural or sequence level.

The assembly and secretion process of bacterial and archaeal S-layers appears multifarious and obviously has evolved independently in some, even closely related microorganisms, such as *Aeromonas* species (Pugsley, 1993; Noonan and Trust, 1995; Thomas and Trust, 1995; Wattiau et al., 1996). By secreting the premature protein into the periplasm, multimerization of the mature proteins in the cytoplasm is prevented.

This mechanism of translocation strongly resembles the formation of type IV pili (Boot and Pouwels, 1996), where pilin precursors are inserted into the cytoplasmic membrane using the Sec translocation pathway (Arts et al., 2007; Francetic et al., 2007). After cleavage of the positively charged signal peptide the highly hydrophobic N-terminus of the mature pilin is exposed and provides a scaffolding interface for the assembly of the entire pilus structure (Bardy et al., 2003; Pohlschröder et al., 2005; Ng et al., 2007; Albers and Pohlschröder, 2009). A similar process was proposed for the formation of the hamus filaments (Probst et al., 2014).

Although the hamus seems to be formed by one major protein, the presence of other proteins involved in its assembly cannot be excluded. Even supposedly simple systems, such as bacterial type IV pili, are usually composed by several pilins and require a certain set of membrane-associated proteins at the basis of the pilus structure (Mattick, 2002; Craig et al., 2004; Nudleman and Kaiser, 2004). Additional hamus-associated proteins could possibly be identified via future co-immunoprecipitation assays, which could then help to understand assembly procedure of the hami and their potential function.

Possible functional traits of the hamus subunit protein were revealed by our combined PSIPRED prediction with a fold recognition search, which revealed three hits, a cytoplasmic response regulator, a xyloglucanase or a human DNA-damage binding protein. Although a functional relation to the DNA damage surveillance proteins serving in the initial detection of UV lesions *in vivo* is difficult to draw for the hamus protein, the first two fold-homologs can be prudently associated with a functional relationship. Xyloglucanases, for instance, hydrolyze polysaccharides from the cellulose microfibrils in plant cell walls (Hayashi and Kaida, 2010). The enzymatic reaction is central to the plant carbon cycle and might also indicate a role of the hami for cell wall degradation and/or carbon metabolism of *Ca*. A. hamiconexum.

Interestingly, the structure of the cytoplasmic response regulator revealed a substantial conformational change on ligand binding and signal transduction, which results in a scissor-like closing. This conformation resembles the barb-like assembly at the hook of the hamus fibril structure, indicative of a signaling role in cell-cell interactions—a possible function of the hami, which had been discussed earlier (Perras et al., 2014). To date it is unclear, whether the hami are involved in other processes apart from cell attachment and biofilm formation. In particular, the anchorage and organization of the hami within the cell wall could not further be resolved using electron cryo-tomography, although it remains without doubt that the hami span both membranes.

Due to the high similarity of the N-terminal amino acid sequence of the hamus subunit to known archaeal S-layer N-termini, one could even hypothesize about a divergent evolution of the hamus subunit protein from ancestral cell surface proteins and thus a conversion of a layered structure toward a filamentous arrangement—concomitant with a loss of the original surface layer and the development of a second membrane. However, it remains elusive if the two membranes of *Ca*. A. hamiconexum are different in their organization and whether a structural and compositional adaptation of the outer membrane has occurred due to the lack of an external S-layer. TOF-SIMS has confirmed that the membranes are mainly composed of diglycosidic diethers (C_20_-C_20_ archaeol and C_20_-C_25_ extended archaeol; Probst et al., 2014). No clear indications of tetraether lipids are observed in the SIMS mass spectra, although trialkyl lipids may be present. Similar to the sodiated series of mono-, di- and tri- glycosylated diether lipid peaks revealed in this study are those of halophilic archaea, *Haloarcula marismortui*, by LC-atmospheric pressure ionization mass spectrometry (de Souza et al., 2009).

In recent years, the field of nanobiotechnology advanced tremendously and is providing an increasing number of strategies to apply natural biomolecules in nanotechnology. For instance, spider silk proved to exhibit extraordinary properties such as strength, elasticity, biocompatibility, and biodegradability and is thus of major interest in nanobiotechnology (Gerritsen, 2002). The spider silk protein could be used in biomedical applications such as coating of implants and drug delivery or scaffolds for tissue engineering (for a review see: Schacht and Scheibel, 2014). However, due to limited availability, experiments with natural spider silk proteins proved complicated (Fox, 1975) and thus the recombinant production of engineered spider silk proteins was pushed forward (Heidebrecht and Scheibel, 2012), including testing of several protein expression systems, with different results in protein yield and property (Chung et al., 2012). Overall, the recombinant expression of the spider silk proteins took researchers decades until a satisfactory result was obtained. Since the hamus subunit protein and the spider silk protein share common features, such as elasticity, robustness and high molecular weight (Moissl et al., 2005; hami: 97 kDa; spider silk protein 250–320 kDa; Sponner et al., 2005; Ayoub et al., 2007), it is not surprising that preliminary overexpression attempts in *Escherichia coli* host strains failed so far. Recombinant expression of the spider silk protein resulted in insufficient yield, as conventional expression strains lacked the capacity of expressing proteins with high molecular weight (Chung et al., 2012). This obstacle was finally overcome by a metabolically engineered *E. coli* expression host, which overexpressed and assembled the silk protein into a strong fiber (Xia et al., 2010). We suggest that a similar approach may be applicable to successful recombinant expression of the hami, which would pave the way for its exploitation in various fields of nanobiotechnology.

The results presented in this communication emphasize the uniqueness of the altiarchaeal hami: their major protein revealed no similarities in sequence and structure to known microbial filament-forming proteins, but showed relationship to archaeal S-layers (in sequence) and beta-sheet protein complexes (in structure), that are widely found in classical macromolecular self-assembled structures. Thus, the hami and the altiarchaeal cell wall (with two membranes and without S-layer) could represent a divergent form of cell organization or even a missing link between euryarchaeal ancestors and the current forms of euryarchaeal life.

### Conflict of interest statement

Patent is pending on the “microbial nano-tool” (Pending European Patent Application No. EP15166985.0). This patent was filed jointly by the AKP, AJP and CME. The authors declare that the research was conducted in the absence of any commercial or financial relationships that could be construed as a potential conflict of interest.
