# Supplementary material for: S-layers at second glance? Altiarchaeal grappling hooks (hami) resemble archaeal S-layer proteins in structure and sequence
Source: Front Microbiol. 2015 Jun 9;6:543. doi: 10.3389/fmicb.2015.00543 (PMC4460559; doi:10.3389/fmicb.2015.00543)
Supplement: Supplementary file 5 [file Presentation1.PPTX]

## Slide 1
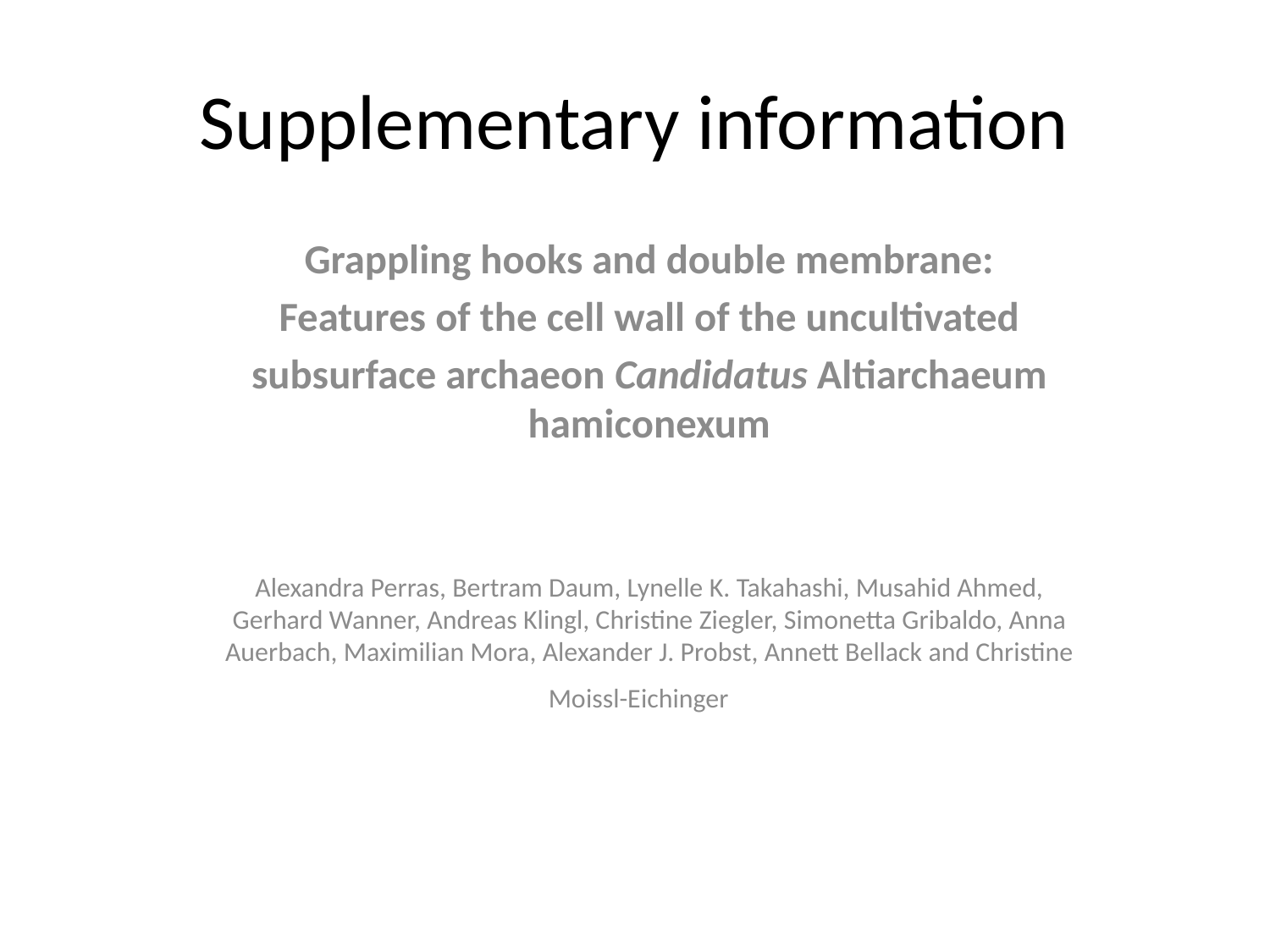

# Supplementary information
Grappling hooks and double membrane:
Features of the cell wall of the uncultivated
subsurface archaeon Candidatus Altiarchaeum hamiconexum
Alexandra Perras, Bertram Daum, Lynelle K. Takahashi, Musahid Ahmed, Gerhard Wanner, Andreas Klingl, Christine Ziegler, Simonetta Gribaldo, Anna Auerbach, Maximilian Mora, Alexander J. Probst, Annett Bellack and Christine Moissl-Eichinger

## Slide 2
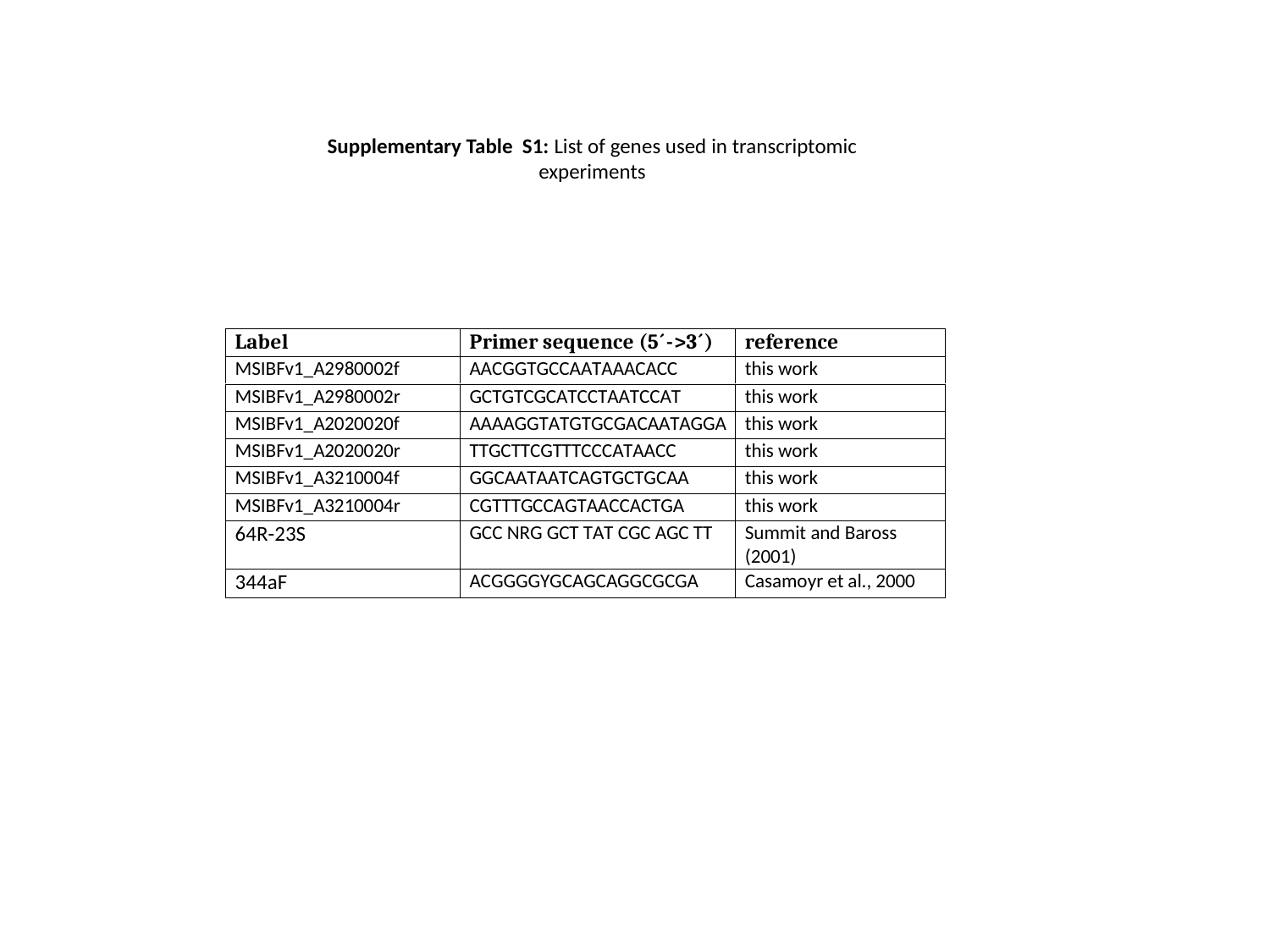

Supplementary Table S1: List of genes used in transcriptomic experiments

## Slide 3
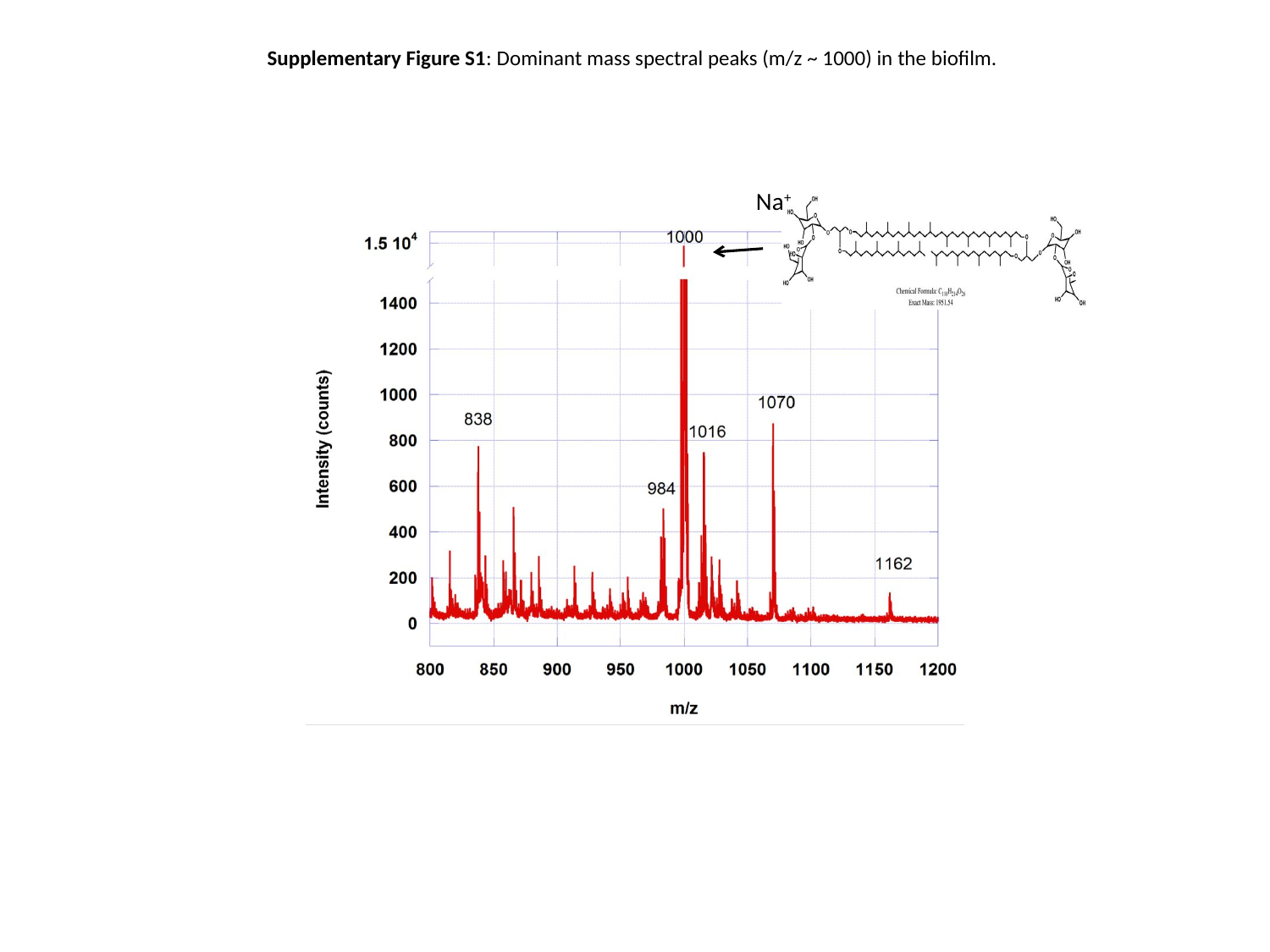

Supplementary Figure S1: Dominant mass spectral peaks (m/z ~ 1000) in the biofilm.
Na+

## Slide 4
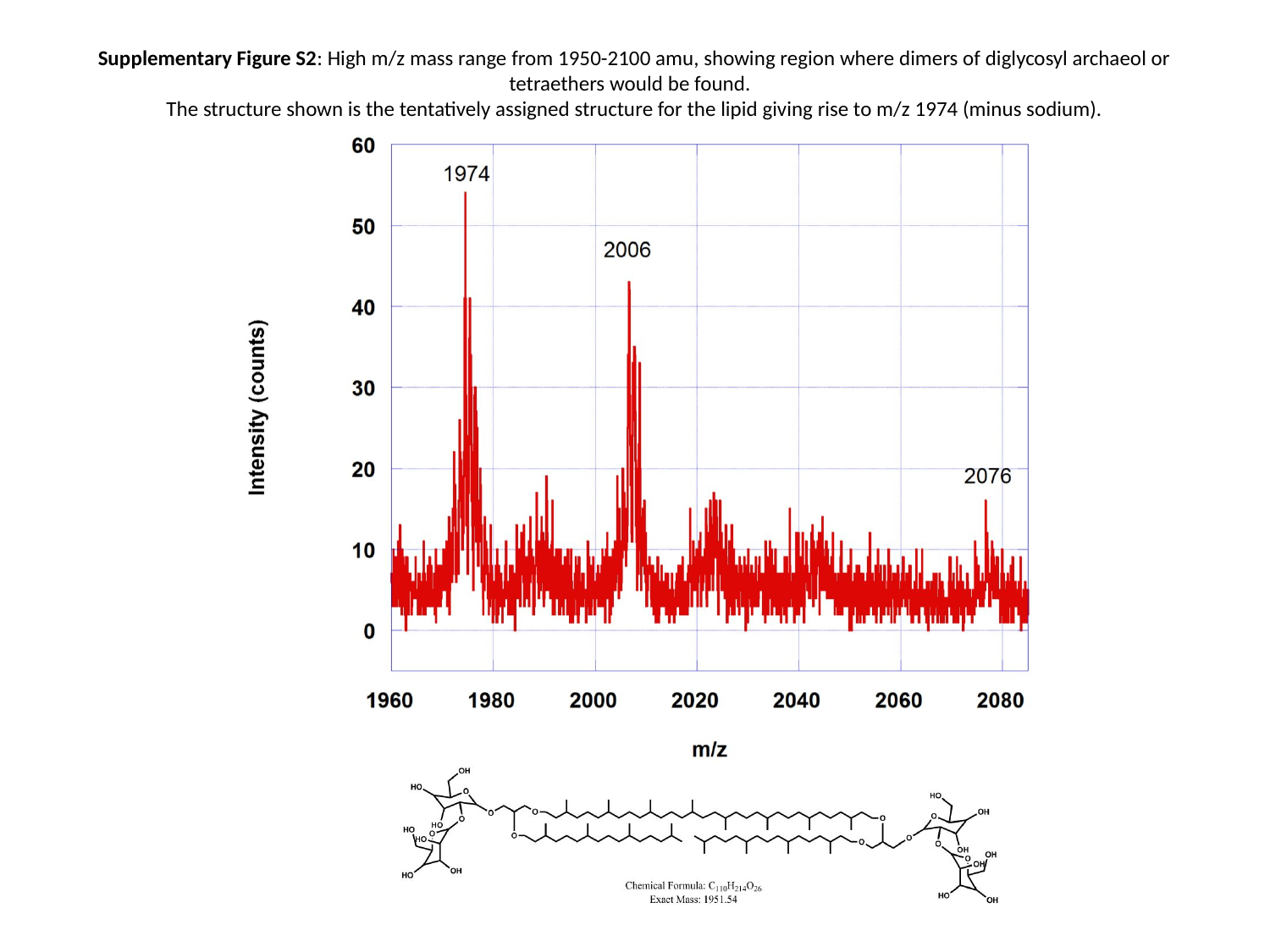

Supplementary Figure S2: High m/z mass range from 1950-2100 amu, showing region where dimers of diglycosyl archaeol or tetraethers would be found.
The structure shown is the tentatively assigned structure for the lipid giving rise to m/z 1974 (minus sodium).

## Slide 5
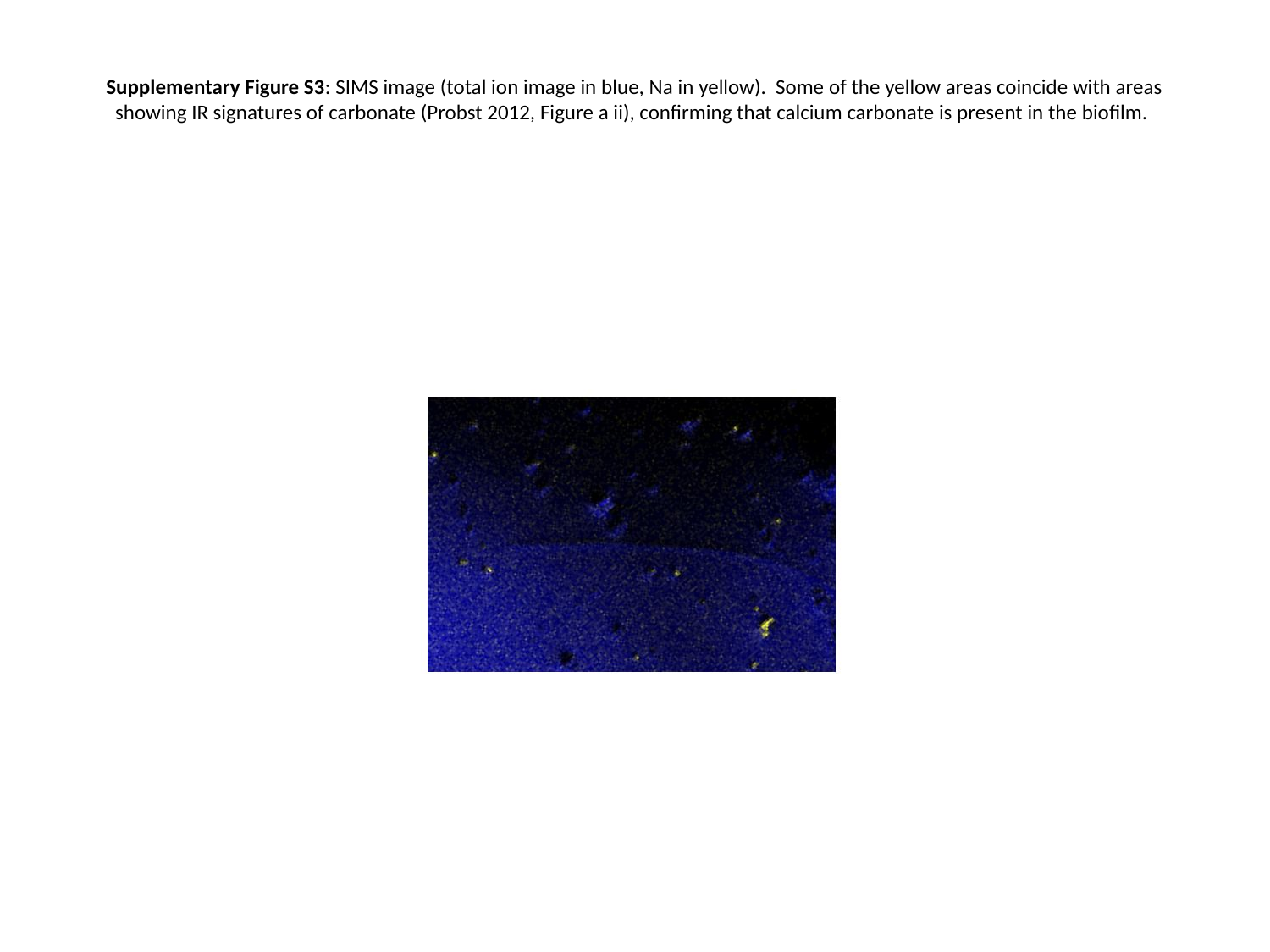

# Supplementary Figure S3: SIMS image (total ion image in blue, Na in yellow). Some of the yellow areas coincide with areas showing IR signatures of carbonate (Probst 2012, Figure a ii), confirming that calcium carbonate is present in the biofilm.

## Slide 6
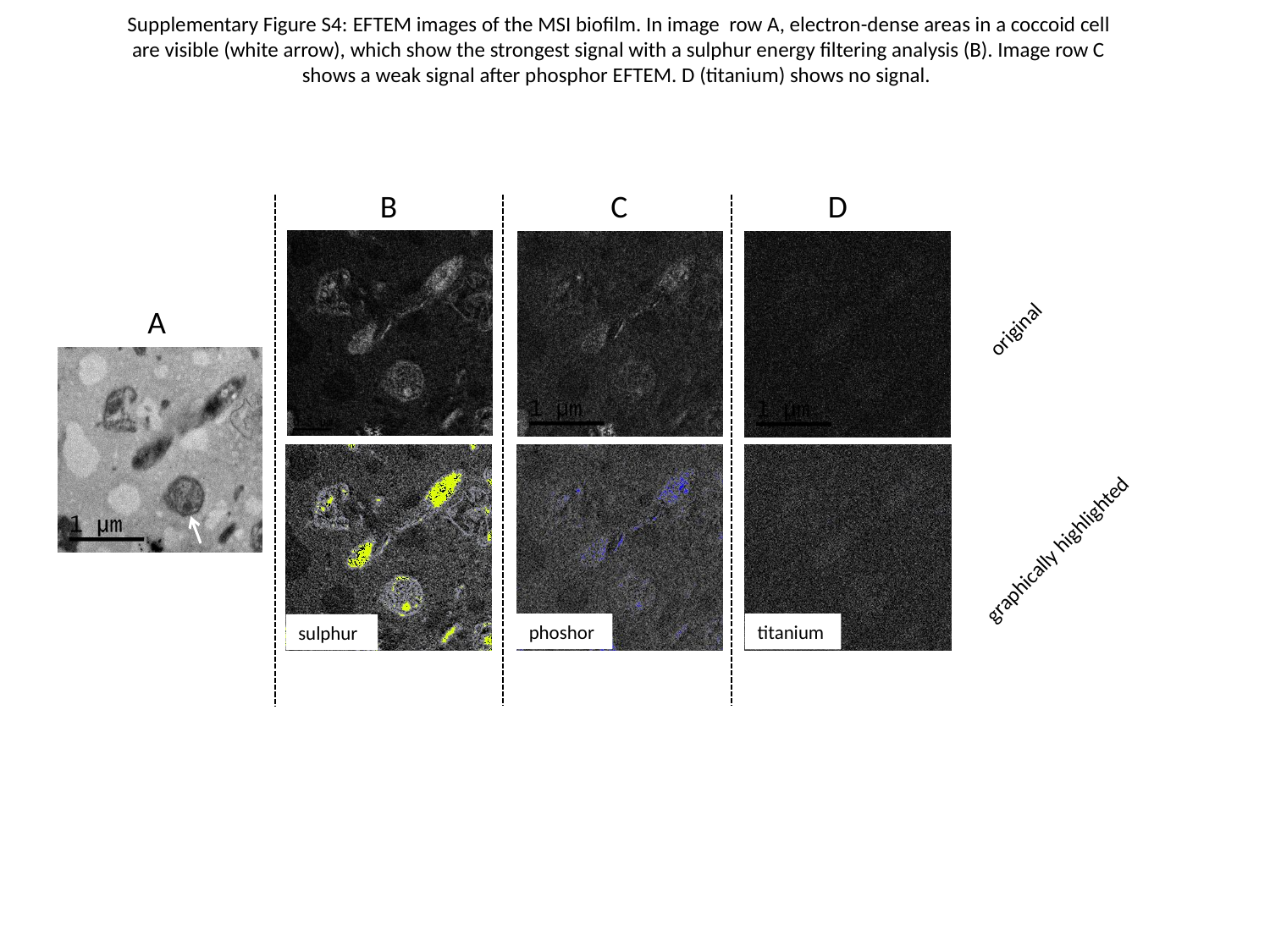

Supplementary Figure S4: EFTEM images of the MSI biofilm. In image row A, electron-dense areas in a coccoid cell are visible (white arrow), which show the strongest signal with a sulphur energy filtering analysis (B). Image row C shows a weak signal after phosphor EFTEM. D (titanium) shows no signal.
B
C
D
A
original
graphically highlighted
phoshor
titanium
sulphur

## Slide 7
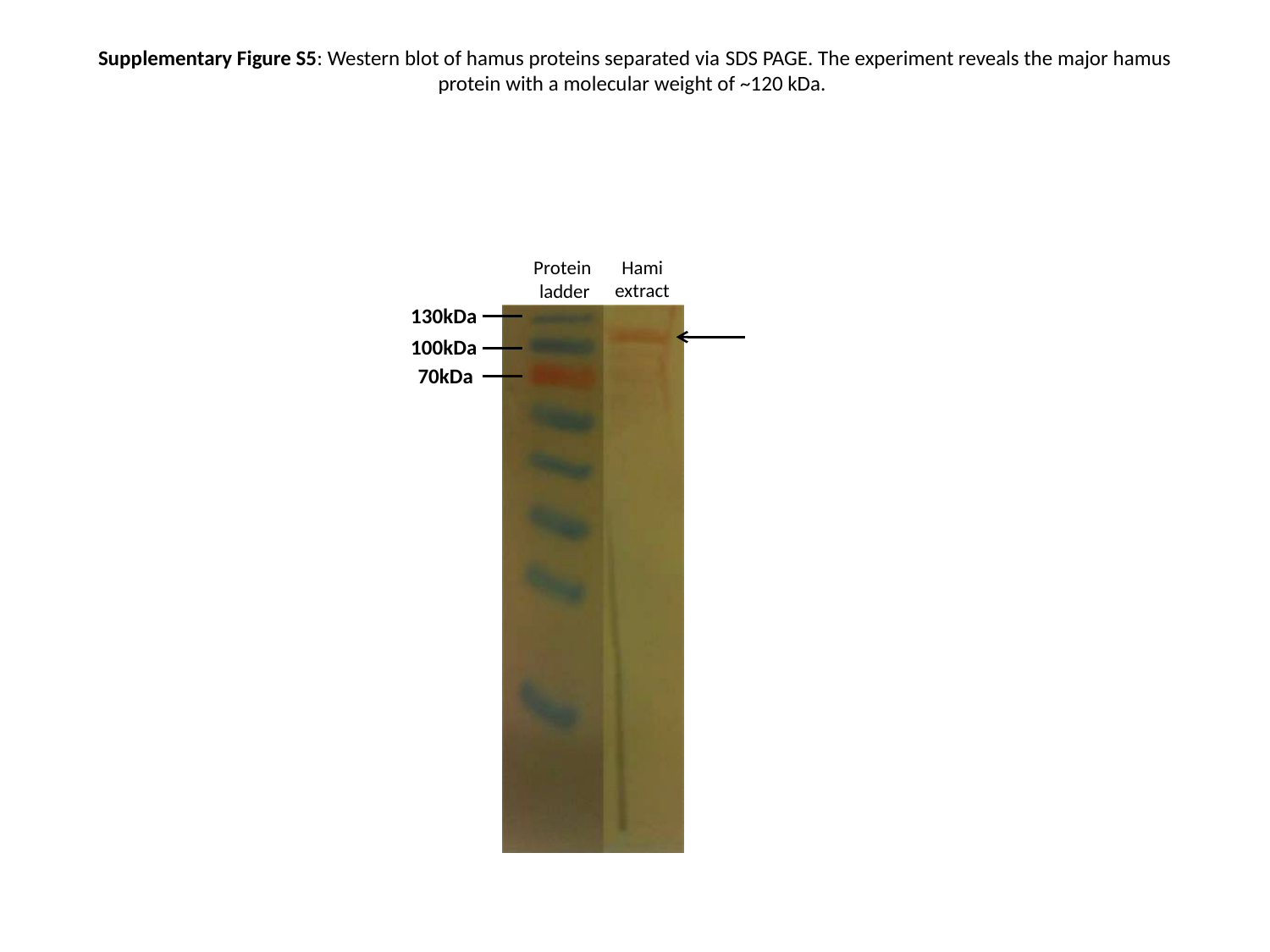

Supplementary Figure S5: Western blot of hamus proteins separated via SDS PAGE. The experiment reveals the major hamus protein with a molecular weight of ~120 kDa.
Hami
extract
Protein
ladder
130kDa
100kDa
 70kDa

## Slide 8
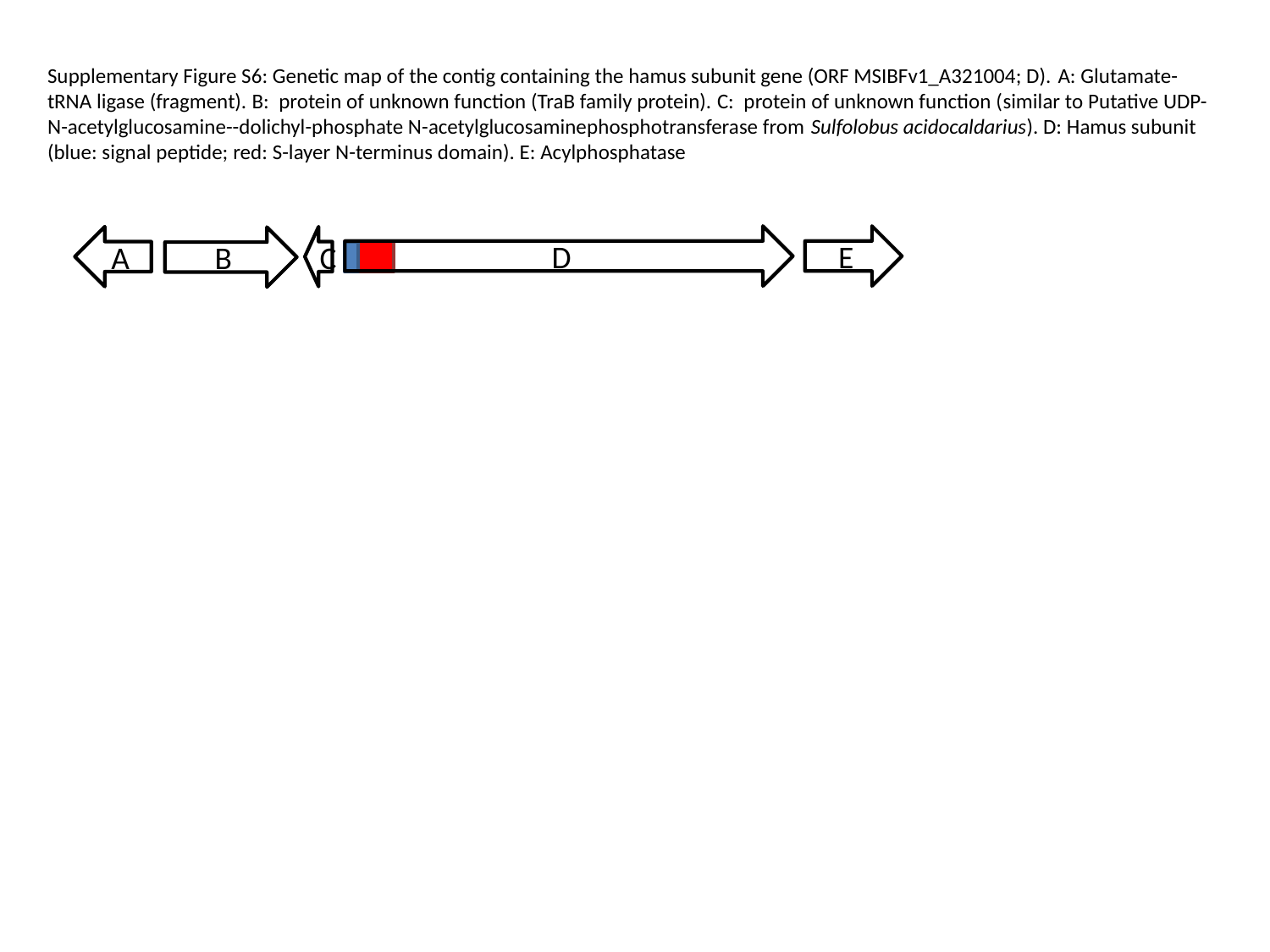

Supplementary Figure S6: Genetic map of the contig containing the hamus subunit gene (ORF MSIBFv1_A321004; D). A: Glutamate-tRNA ligase (fragment). B: protein of unknown function (TraB family protein). C: protein of unknown function (similar to Putative UDP-N-acetylglucosamine--dolichyl-phosphate N-acetylglucosaminephosphotransferase from Sulfolobus acidocaldarius). D: Hamus subunit (blue: signal peptide; red: S-layer N-terminus domain). E: Acylphosphatase
D
E
A
C
B

## Slide 9
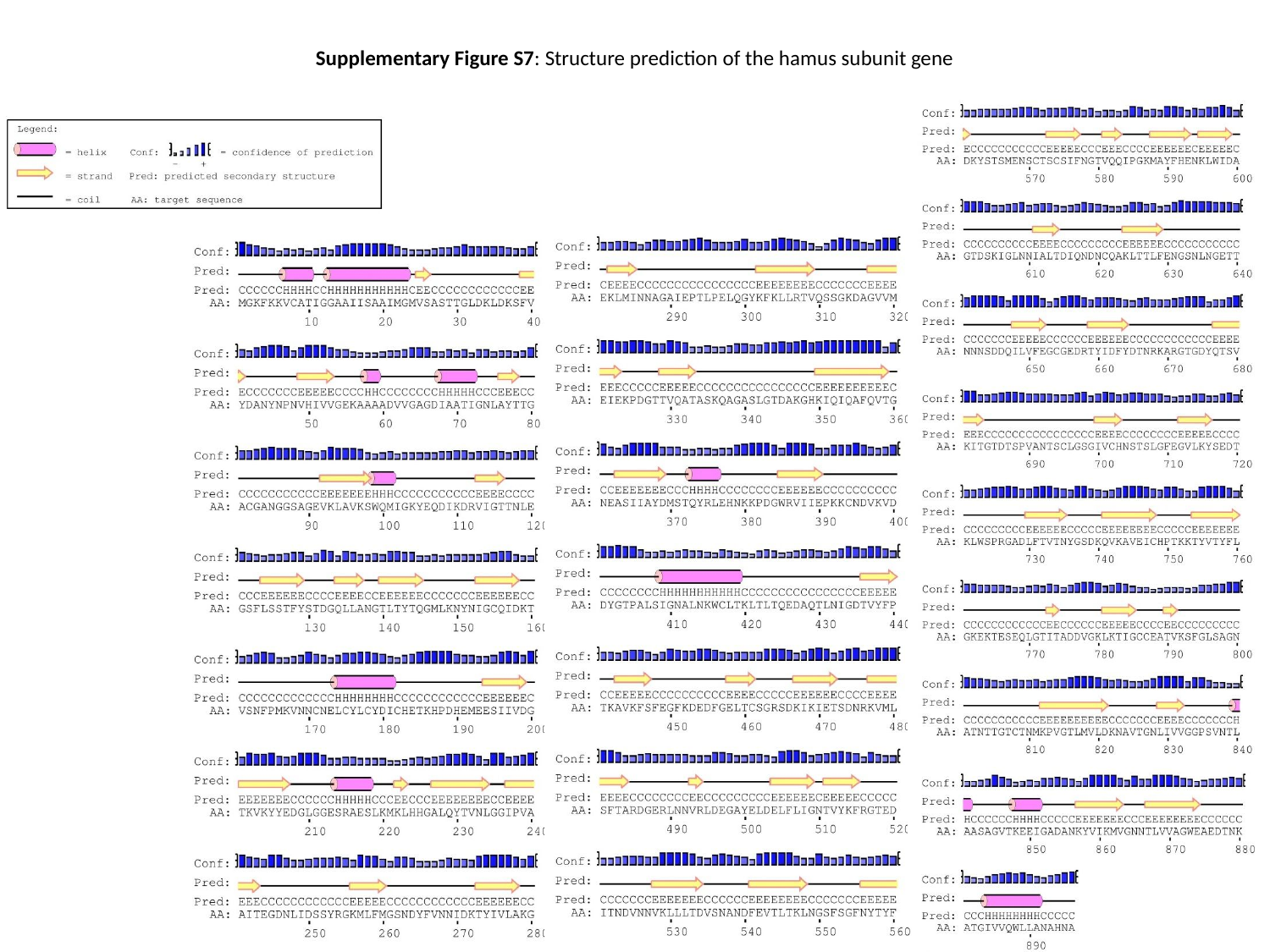

Supplementary Figure S7: Structure prediction of the hamus subunit gene

## Slide 10
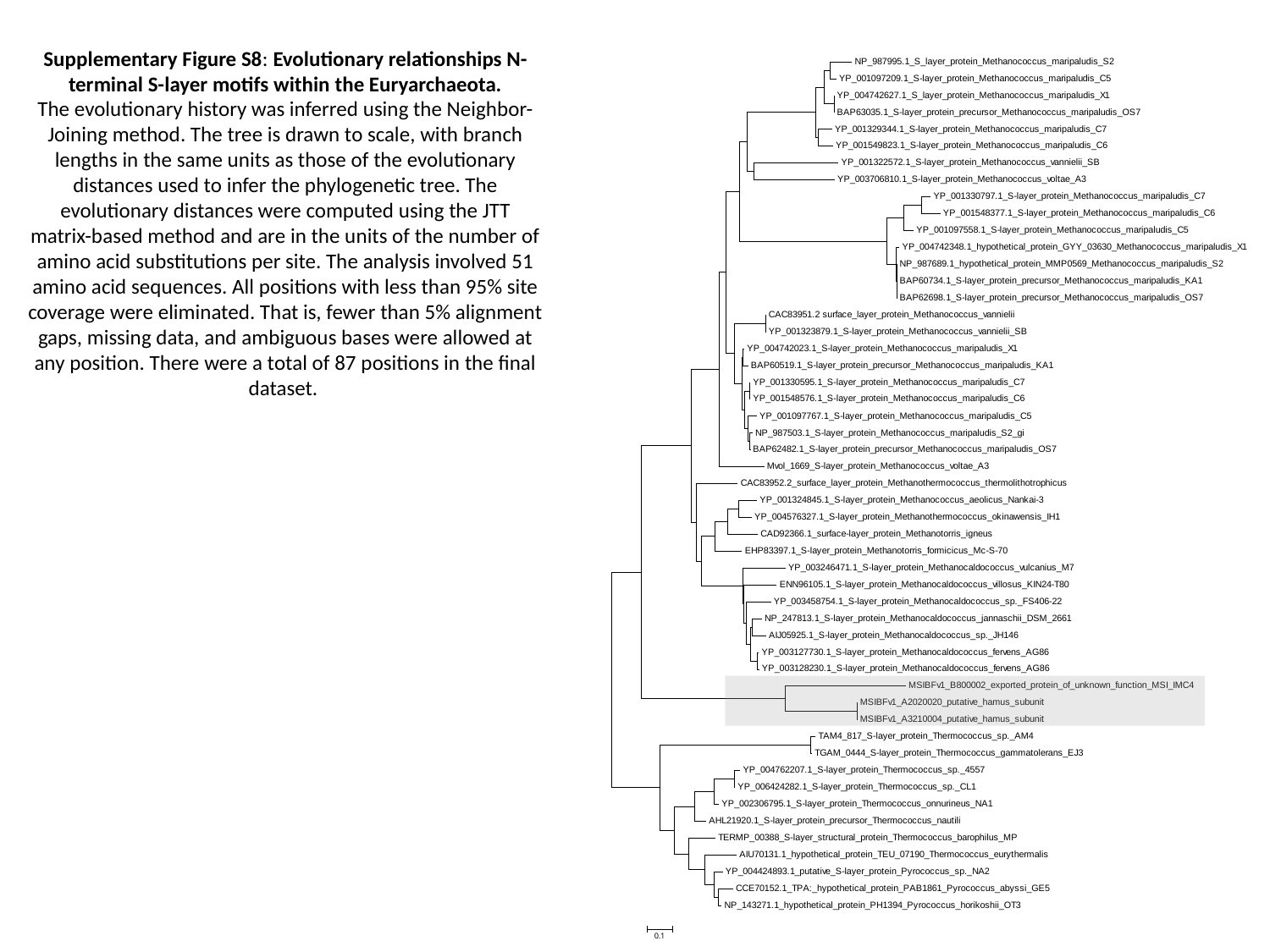

Supplementary Figure S8: Evolutionary relationships N-terminal S-layer motifs within the Euryarchaeota.
The evolutionary history was inferred using the Neighbor-Joining method. The tree is drawn to scale, with branch lengths in the same units as those of the evolutionary distances used to infer the phylogenetic tree. The evolutionary distances were computed using the JTT matrix-based method and are in the units of the number of amino acid substitutions per site. The analysis involved 51 amino acid sequences. All positions with less than 95% site coverage were eliminated. That is, fewer than 5% alignment gaps, missing data, and ambiguous bases were allowed at any position. There were a total of 87 positions in the final dataset.

## Slide 11
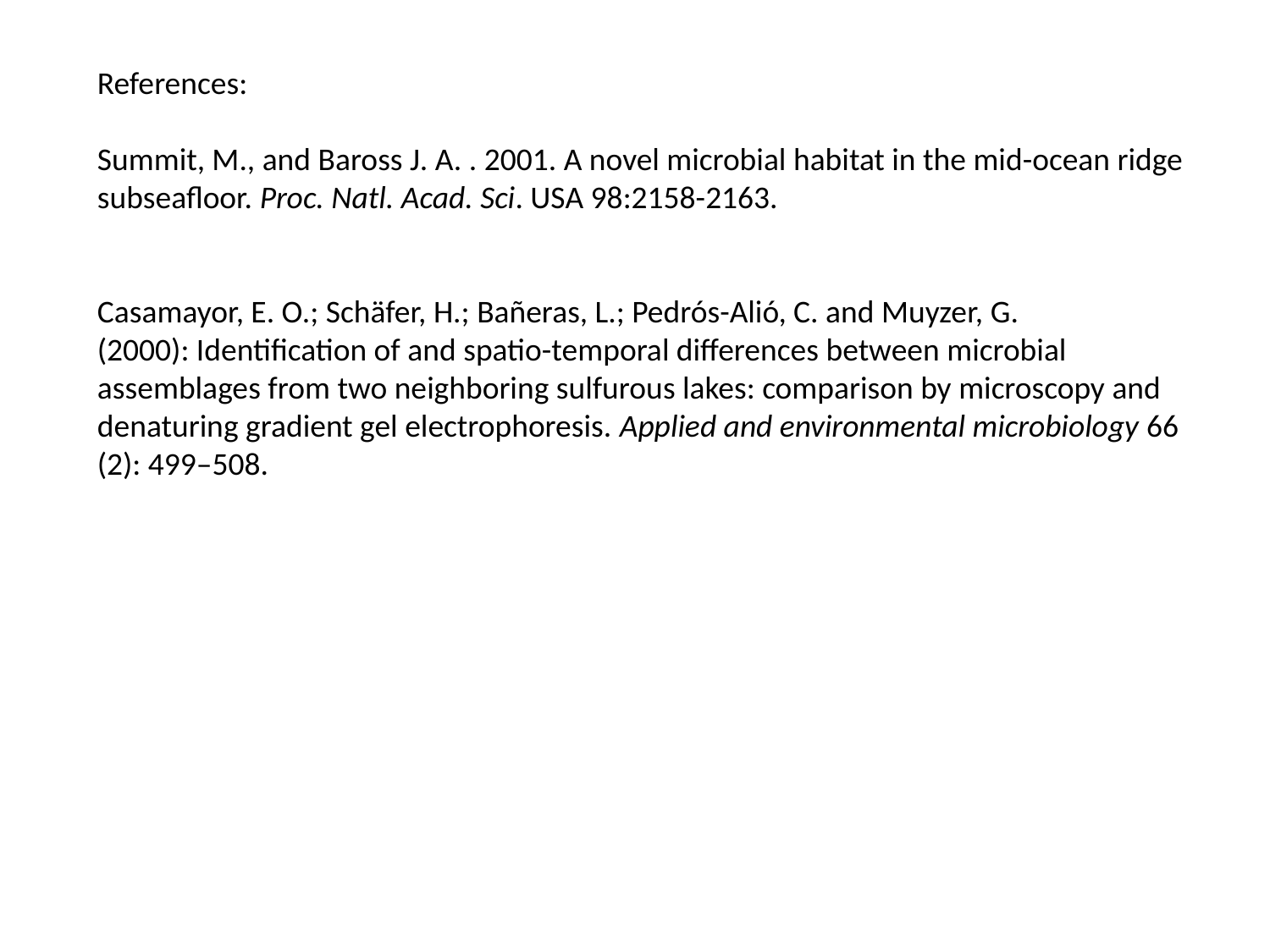

References:
Summit, M., and Baross J. A. . 2001. A novel microbial habitat in the mid-ocean ridge subseafloor. Proc. Natl. Acad. Sci. USA 98:2158-2163.
Casamayor, E. O.; Schäfer, H.; Bañeras, L.; Pedrós-Alió, C. and Muyzer, G.
(2000): Identification of and spatio-temporal differences between microbial assemblages from two neighboring sulfurous lakes: comparison by microscopy and denaturing gradient gel electrophoresis. Applied and environmental microbiology 66 (2): 499–508.
